# Fatal *Rickettsia parkeri* Rickettsiosis in Immunocompromised Pediatric Patient, Georgia, USA, 2025

**DOI:** 10.3201/eid3211.261174

**Published:** 2026-11

**Authors:** Stephanie Bellman, Thomas G. Fox, Sunil S. Raikar, Daniel John Bergsagel, Meredith Steuer, Kiran B. Hebbar, Jennifer Kasten-Metz, Sandor E. Karpathy, Christopher D. Paddock, Kristy O. Murray

**Affiliations:** Children’s Healthcare of Atlanta, Atlanta, Georgia, USA (S. Bellman, T.G. Fox, S.S. Raikar, D.J. Bergsagel, M. Steuer, K.B. Hebbar, K.O. Murray); Emory University School of Medicine, Atlanta (S. Bellman, T.G. Fox, S.S. Raikar, D.J. Bergsagel, M. Steuer, K.B. Hebbar, K.O. Murray); Centers for Disease Control and Prevention, Atlanta (J. Kasten-Metz, S.E. Karpathy, C.D. Paddock)

**Keywords:** vector-borne infections, bacteria, case report, *Rickettsia parkeri*, spotted fever group, immunocompromised, fatality, tick-borne infections, USA

## Abstract

We describe a fatal *Rickettsia parkeri* rickettsiosis case in a 3-year-old boy in Georgia, USA, receiving chemotherapy for B-cell acute lymphoblastic leukemia, hospitalized with febrile neutropenia. Plasma microbial cell-free DNA sequencing confirmed the diagnosis. This report highlights the need to maintain suspicion for tickborne diseases in immunocompromised hosts in some settings.

*Rickettsia parkeri* rickettsiosis is a mild to moderately severe spotted fever rickettsiosis (SFR) characterized by fever, myalgias, headache, rash, and, commonly, an eschar at the site of tick attachment (*1*). Since 2020, nearly 5,000 cases of SFR have been reported in the United States, including infections with *R. parkeri* (*2*). A systematic review of 77 *R. parkeri* rickettsiosis cases found that only 9% (16% in confirmed cases) required hospitalization and only 7% (3% in confirmed cases) occurred in children (*3*). Doxycycline is the treatment of choice for all rickettsial infections. We describe a fatal case of *R. parkeri* infection in an immunocompromised child in Georgia, USA.

## The Study

A 3-year-old boy from southwestern Georgia receiving chemotherapy for high-risk B-cell acute lymphoblastic leukemia was admitted to the hospital in late July 2025 with febrile neutropenia. At admission, he had profound neutropenia and thrombocytopenia (leukocyte count 110/µL, absolute neutrophil count 20/µL, platelets 19,000/µL). He was on the second month of delayed intensification chemotherapy as part of a high-risk B-cell acute lymphoblastic leukemia regimen and had received cyclophosphamide, cytarabine, and 6-thioguanine 2 weeks before admission. His examination was unremarkable except for 2 pink papulonodules with central eschars on the left upper extremity (Figure 1, panel A). He was started on empiric cefepime therapy after blood cultures were obtained.

**Figure 1:**
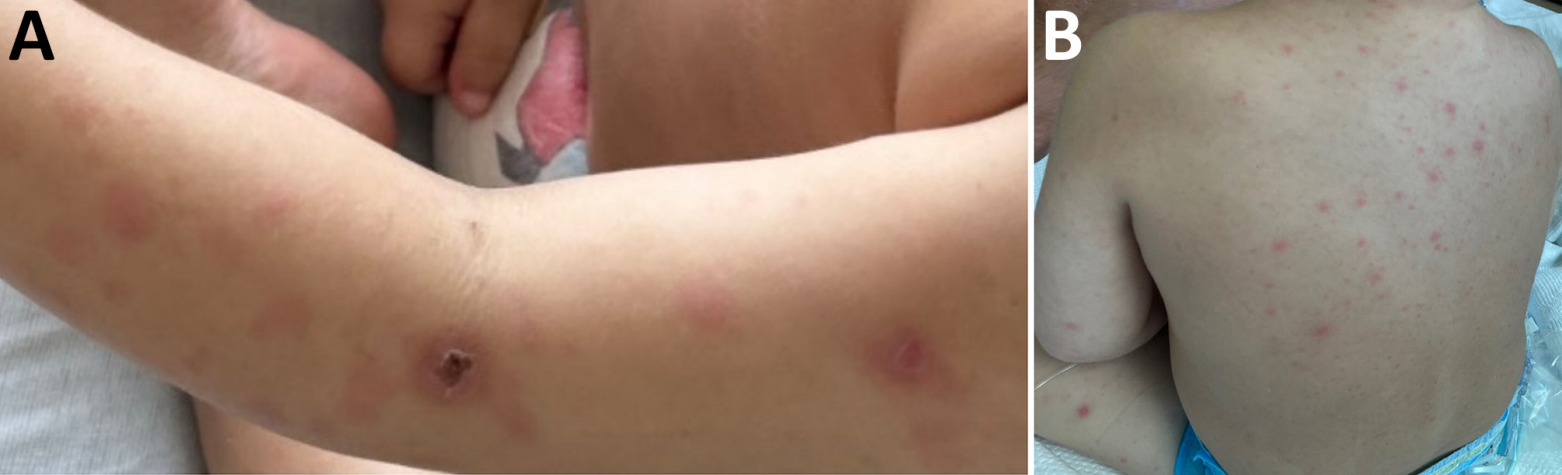
Photographs of rash from study of fatal *Rickettsia parkeri* rickettsiosis in immunocompromised pediatric patient, Georgia, USA, 2025. A) Eschar on left upper extremity; B) rash on patient’s torso.

On the second and third day of hospitalization, a rash developed that began with small pink macules on the upper back and arms (Figure 1, panel B). Over the next 3 days, the rash progressed to numerous pink macules, some with overlying vesicles involving the bilateral upper and lower extremities, face, and trunk. Upon further questioning, his parents reported that the lesions on his left arm had been present for about 1 month before hospitalization and had been draining clear fluid. They attributed those lesions to suspected insect bites because he frequently played in the grass around their suburban home, but they did not recall seeing any insects.

He remained febrile despite cefepime therapy and had persistent pancytopenia and unmeasurable lymphocyte count; blood culture results were negative. Micafungin and acyclovir were initiated to target potential disseminated infection with *Candida* or herpes viruses. PCR testing for herpes simplex virus and varicella zoster virus from an unroofed vesicle returned negative results. Computed tomography with intravenous contrast of the lungs showed minimal ground glass opacities; abdominal ultrasound revealed hepatomegaly, trace ascites, and thickened and inflamed small bowel loops consistent with neutropenic enteritis.

On hospital day 7, he developed hypotension and respiratory failure and was transferred to the intensive care unit, requiring bilevel positive airway pressure. Antimicrobial treatment was changed to meropenem, vancomycin, amphotericin B, isavuconazole, and doxycycline. He continued to have hemodynamic deterioration and worsening hypoxia. During intubation, he suffered a 20-minute cardiac arrest requiring chest compressions before return of spontaneous circulation, but unfortunately he later developed fixed and dilated pupils. The decision was made to withdraw life-sustaining therapies, and he died on hospital day 8. The next day, a sample submitted on hospital day 7 for plasma microbial cell-free DNA sequencing (Karius, https://kariusdx.com) returned positive results for *R. parkeri* (8,374 molecules/100 nL).

Confirmatory laboratory testing was performed by the Centers for Disease Control and Prevention (Atlanta, GA, USA). DNA was extracted from the residual plasma specimen and was found positive by real-time PCR targeting a 96-bp segment of the *omp*B gene specific for *R. parkeri* (*4*). Formalin-fixed, paraffin-embedded sections of the skin biopsy specimen were stained by an immunoalkaline phosphatase technique for multiple species of spotted fever group *Rickettsia*, including *R. parkeri*, yielding abundant staining for rickettsiae and rickettsial antigens in the lumina and endothelium of vessels and extension into adjacent dermal tissue (Figure 2) (*1*).

**Figure 2:**
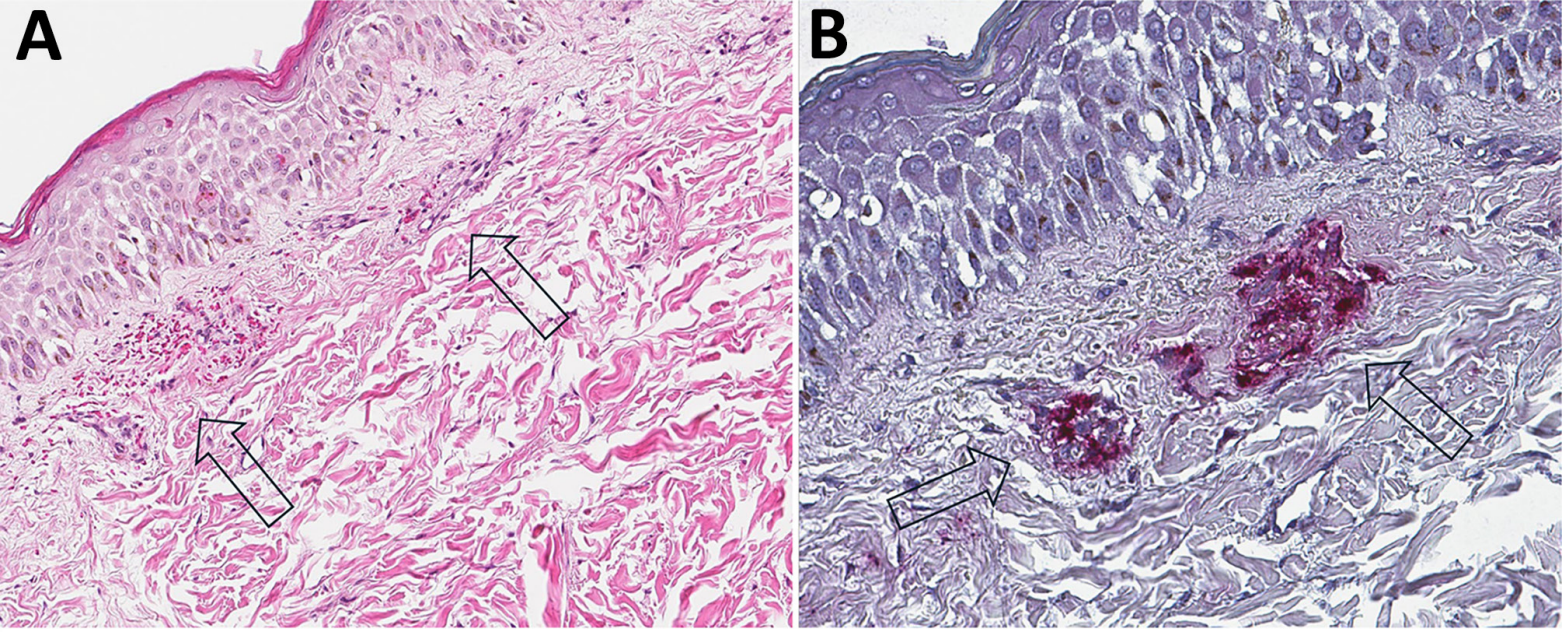
Immunohistochemistry and skin pathology from study of fatal *Rickettsia parkeri* rickettsiosis in immunocompromised pediatric patient, Georgia, USA, 2025. A) Punch biopsy specimen obtained from a rash lesion of the lower extremity of the patient demonstrating mild superficial dermal edema and small perivascular hemorrhages (arrows) without distinct inflammatory cell infiltrates. Hematoxylin and eosin stain; original magnification ×62.5. B) Immunohistochemical staining using an antibody reactive with spotted fever group *Rickettsia* spp., including *Rickettsia parkeri*, shows abundant staining of rickettsiae and rickettsial antigens in the lumens of the vessels, with invasion through the endothelium into the surrounding tissue (arrows). Immunoalkaline phosphatase with naphthol fast red and hematoxylin counterstain; original magnification ×62.5.

## Conclusions

In this article, we describe a fatal case of *R. parkeri* rickettsiosis in a young, immunocompromised child. There is 1 known case of *R. parkeri* infection in an immunocompromised patient: a kidney transplant recipient who developed a moderately severe *R. parkeri* infection shortly after transplantation characterized by fever, diffuse rash, arthralgias, elevated inflammatory markers, and mildly elevated transaminases, which resolved after treatment with doxycycline (*5*). 

Multiple factors contributed to the delay in recognition of SFR in this patient. *R. parkeri* infection is more commonly diagnosed in adults >40 years of age (*2*,*6*). In addition, hospital admission for febrile neutropenia is common during the stage of chemotherapy this child was receiving. A comprehensive study of >600 children with acute lymphoblastic leukemia showed that 72.9% of patients receiving high-risk delayed intensification were hospitalized at least once (*7*). The initial antibiotic for febrile neutropenia is typically chosen to target gram-negative bacteria, with a fourth-generation cephalosporin (e.g., cefepime) administered according to institutional guidelines (*8*). Persistent fevers and clinical deterioration led to the expansion of antibiotic treatment, covering gram-positive bacteria and fungal pathogens, a common strategy used in febrile neutropenia without confirmatory infectious results. Unfortunately, given the treatment phase and the reason for admission, recognizing a tickborne rickettsial infection was challenging. Clinicians might not consider those infections in patients with common syndromes like febrile neutropenia. Advanced diagnostic tools, such as unbiased cell-free DNA sequencing (e.g., Karius Spectrum), can expand our ability to detect fastidious or difficult to diagnose pathogens (*9*). Such tools have been increasingly used to detect rickettsial species (*5*,*10*,*11*), although the outsourcing of the test takes additional time to obtain a result, which can delay pathogen-targeted treatment.

Although a history of presumed insect bites was reported, no tick or insect was ever visualized on the child. Diagnosis of tickborne infection is often difficult without known tick exposure. *R. parkeri* is transmitted by the Gulf Coast tick, *Amblyomma maculatum*, the range for which includes the part of southwestern Georgia where this patient lived (*12*). Adult ticks are most active during July and August near the Gulf Coast (*13*), coinciding with the timing of this case.

The manifestation of *R. parkeri* rickettsiosis in this immune-suppressed patient was atypical for multiple reasons and contributed to the unusual severity of disease. The rash associated with *R. parkeri* consists typically of scattered pink macules with overlying vesicles (*1*). This patient’s rash was more extensive than what is typically reported, including facial lesions. Furthermore, real-time PCR conducted at Centers for Disease Control and Prevention detected a high concentration of *R. parkeri* rickettsia in the plasma, and pathology examination found a high concentration of rickettsial antigen stain in and around dermal vessels. In vitro studies of rickettsial infection have demonstrated the importance of CD4 and CD8 T-cells, especially highlighting the necessity of CD8 T-cells in successfully eliminating the pathogen (*14*). The patient we describe had profound lymphopenia from his underlying leukemia and chemotherapy, which likely contributed to increased disease severity. In addition, a study in mice infected with *R. parkeri* demonstrated lethality with high inoculation dose (*15*), indicating that, whereas the infection is typically mild, high pathogen concentrations could be linked to death. On the basis of this case and the previous report in a kidney transplant patient (*5*), *R. parkeri* rickettsiosis represents a larger threat to immunocomprimised patients than previously thought.

This case highlights a rare but potentially serious tickborne infection in immunocompromised hosts and the importance of recognizing SFR in patients not typically considered at high risk. Clinicians should consider *R. parkeri* rickettsiosis in patients seeking care for fevers and eschar lesions who reside in or travel to areas where *A. maculatum* ticks exist, particularly during summer months.
